# Cardiac computed tomography-derived coronary artery volume to myocardial mass for the prediction of risk stratification for acute coronary syndrome

**DOI:** 10.3389/fcvm.2025.1449148

**Published:** 2025-02-13

**Authors:** E. Ding, Liang Chen, Xiao-yu Wei, De-shu You, Chang-jie Pan

**Affiliations:** Department of Radiology, The Affiliated Changzhou No.2 People’s Hospital of Nanjing Medical University, Changzhou, China

**Keywords:** coronary computed tomography angiography, coronary artery lumen volume to myocardial mass, acute coronary syndrome, stable angina pectoris, GRACE risk score

## Abstract

**Purpose:**

The study aimed to assess various characteristics of coronary computed tomography angiography (CCTA) in patients presenting with suspected coronary artery disease (CAD). Additionally, the research sought to investigate the predictive value of the coronary artery volume to myocardial mass (V/M) derived from CCTA in risk stratification for patients with acute coronary syndrome (ACS) and to determine the relationship between the V/M ratio and the Global Registry of Acute Coronary Events (GRACE) risk score in ACS.

**Methods:**

This was a single-center, retrospective study. The magnitude of V/M was investigated in patients with ACS (*n* = 168), stable angina pectoris (SAP) (*n* = 160), and healthy controls (*n* = 122) among 450 patients with suspected CAD who did not require urgent angiography. Patients underwent CCTA for 0.5–6 months (median 3.3 months) before the SAP and ACS event. All patients underwent invasive coronary angiography (ICA) at the time of the SAP and ACS event. The Mantel test was used to assess the factors influencing risk stratification in CAD. Receiver Operating Characteristic (ROC) curve analysis was used to assess the accuracy of the V/M ratio in predicting ACS. Pearson correlation analysis was utilized to analyze the correlation between V/M and GRACE risk score, and independent predictors of high GRACE risk score were screened using univariate and multivariate logistic regression analysis.

**Results:**

The Mantel test analysis shows that the key factors of ACS were left ventricle myocardial mass (M), V/M, and coronary CT angiography-derived fractional flow reserve (FFR_CT_) (*p* < 0.01). The V/M ratio in ACS and SAP was significantly lower than in controls (21.7 ± 6.96, 31.0 ± 9.90, vs. 43.3 ± 11.50 mm^3^/g; *p* < 0.001). Lower V/M ratios were found with the progression of CAD from SAP to unstable angina pectoris (UAP) to acute myocardial infarction (AMI) (17.8 ± 5.30, 24.3 ± 6.70, vs. 31.0 ± 9.90 mm^3^/g; *p* < 0.001). ROC analysis shows that V/M outperformed FFR_CT_, % DS in predicting ACS [AUC: 0.78 [95% CI: 0.74–0.83] vs. 0.74 [95% CI: 0.69–0.79], 0.60 [95% CI: 0.53–0.64]], and the combined AUC of the three increased significantly, reaching 0.80 [95%(CI): 0.76–0.85]. Furthermore, in the subgroup of ACS patients, the results of Pearson correlation analysis shows that the GRACE risk score of ACS patients was significantly negatively correlated with the V/M ratio and V/M was found to be an independent predictor of GRACE risk score >140 (*p* < 0.001).

**Conclusions:**

The V/M ratio is valuable for stratified risk prediction of ACS and is independently associated with the GRACE risk score.

## 1 Introduction

The size of the coronary luminal volume is directly correlated to blood flow and myocardial mass, as has been confirmed by both animal and human studies (1, 2). The recently reported *post-hoc* analysis of the NeXt sTeps (NXT) trial (NCT01757678) proposed V/M, a metric based on cardiac computed tomography-derived coronary artery volume to left ventricle myocardial mass. V/M has proven to be a valuable tool for quantifying potential physiological imbalances between blood supply and myocardial demand (3). Additionally, the GRACE risk score, initially established to predict short-term (in-hospital) mortality in patients with ACS (4), is still one of the most robust risk-predicting models in the setting of ACS among numerous risk-scoring systems.

For individuals with chronic CAD, the progressive narrowing of coronary arteries due to atherosclerosis often precipitates a mismatch between blood supply and oxygen demand in the heart, leading to symptoms of myocardial ischemia. These associations strongly indicate a distinct mechanistic role for the V/M ratio in driving the progression of ACS. Advancements in non-invasive imaging modalities could enhance the early detection and risk stratification of individuals predisposed to ACS. While invasive coronary angiography (ICA) remains the gold standard for delineating coronary heart disease (5), characterized by its precision in assessing coronary anatomy, it may fall short in detecting diffuse atherosclerotic plaque in non-obstructive coronary artery disease. Conversely, myocardial perfusion imaging, a non-invasive modality, can quantify ischemia but lacks the direct visualization of coronary anatomy (6). In other words, current assessment practices for coronary artery disease often evaluate coronary artery anatomy and myocardium independently. This could be enhanced by integrating V/M data, as this integration enhances the anatomical and functional insights obtained from CCTA, FFR_CT_, and computed tomography myocardial perfusion (6). Such a comprehensive approach promises to refine risk stratification in patients with stable CAD and recent ACS, potentially influencing clinical diagnosis and management paradigms.

Over the past few years, several studies have investigated the role of V/M in conditions affecting the coronary artery tree and myocardium, or both. However, further investigation is required to fully elucidate the interplay between V/M and recent ACS. Accordingly, this study aimed to (1) assess the level in patients with and without CAD and compare the V/M ratio across the entire spectrum of patients with CAD; (2) assess the accuracy of the V/M ratio to predict the presence of ACS; (3) determine the relationship between the V/M ratio and GRACE risk score in ACS.

## 2 Material and methods

The present study was approved by the ethics committee of The Changzhou No. 2 People's Hospital affiliated with Nanjing Medical University. Informed consent was not required because of the retrospective study design.

### 2.1 Patients

Consecutive patients clinically suspected of CAD admitted to Changzhou No. 2 People's Hospital affiliated with Nanjing Medical University between January 2020 and September 2023 were included in this retrospective analysis. Inclusion criteria were the following: (1) 18–80 years old; (2) the undertaking of CCTA within a six-month interval preceding ICA; (3) the confirmation of stenosis exceeding 50% in one or more vessels via coronary angiography. Exclusion criteria were: (1) previous history of myocardial infarction or coronary revascularization; (2) secondary myocardial infarction caused by other common diseases such as myocarditis; (3) severe arrhythmia or renal insufficiency; (4) anatomical heart or coronary artery variations; and (5) poor quality coronary CTA images. The indications for CCTA are as follows: (1) Patients suspected of having coronary artery disease, particularly those in high-risk groups, including individuals with hypertension, diabetes mellitus, hyperlipidemia, a family history of coronary artery disease, and other risk factors such as smoking. (2) Abnormal results on exercise electrocardiography. (3) Patients with unexplained chest pain. (4) Patients without obvious arrhythmias.

SAP was defined as no episodes of angina at rest but angiographically documented organic stenosis of >50% in at least one of the major coronary arteries. The stratification of ACS adhered to the classifications set forth by the European Society of Cardiology and the American College of Cardiology/American Heart Association guidelines, delineating ACS into ST-segment elevation ACS, non-ST-segment elevation ACS, and UAP (7). In the present study, the median time-to-interval between CCTA and the occurrence of clinical events (SAP and ACS) ranged from 0.5–6 months, with a median of 3.3 months. The final cohort comprised 450 participants: 168 patients diagnosed with ACS, 160 patients with SAP, and 122 individuals presenting with noncoronary heart disease and normal coronary angiography during the same period as the control group. The healthy controls were well-matched to the ACS and SAP patients in terms of age, sex, smoking status, cardiovascular family history, and dyslipidemia. The patients with ACS were categorized into sub-groups based on their GRACE risk score. The low-intermediate risk group (GRACE risk score ≦ 140) comprised 138 patients, while the high-risk group (GRACE risk score >140) consisted of 30 patients.

### 2.2 CCTA image acquisition

CCTA was conducted on Siemens' third-generation dual-source CT system (SOMATOM definition force; Siemens AG, Munich, Germany). Before the scan, patients rested for 15 min and, if needed, received up to 30 mg intravenous metoprolol to lower their heart rate below 65 bpm. Sublingual nitroglycerin was not used before the scan. The scan used prospective electrocardiogram gating, covering the area from the superior sternal fossa to the diaphragmatic surface of the heart. Scan settings included a 100 kV tube voltage, 228–300 mA tube current, 0.25 s rotation time, 0.75 mm layer thickness, 75% RR cycle x-ray window, 0.50 mm reconstruction interval, and 512 × 512 display matrix. An iodixanol injection (100 ml: 32 g; Qing Liming, NJCTTQ, China) was administered through the middle elbow vein at 60–80 ml volume and 5–6 ml/s speed.

### 2.3 Volume to mass analysis

**Figure 1:**
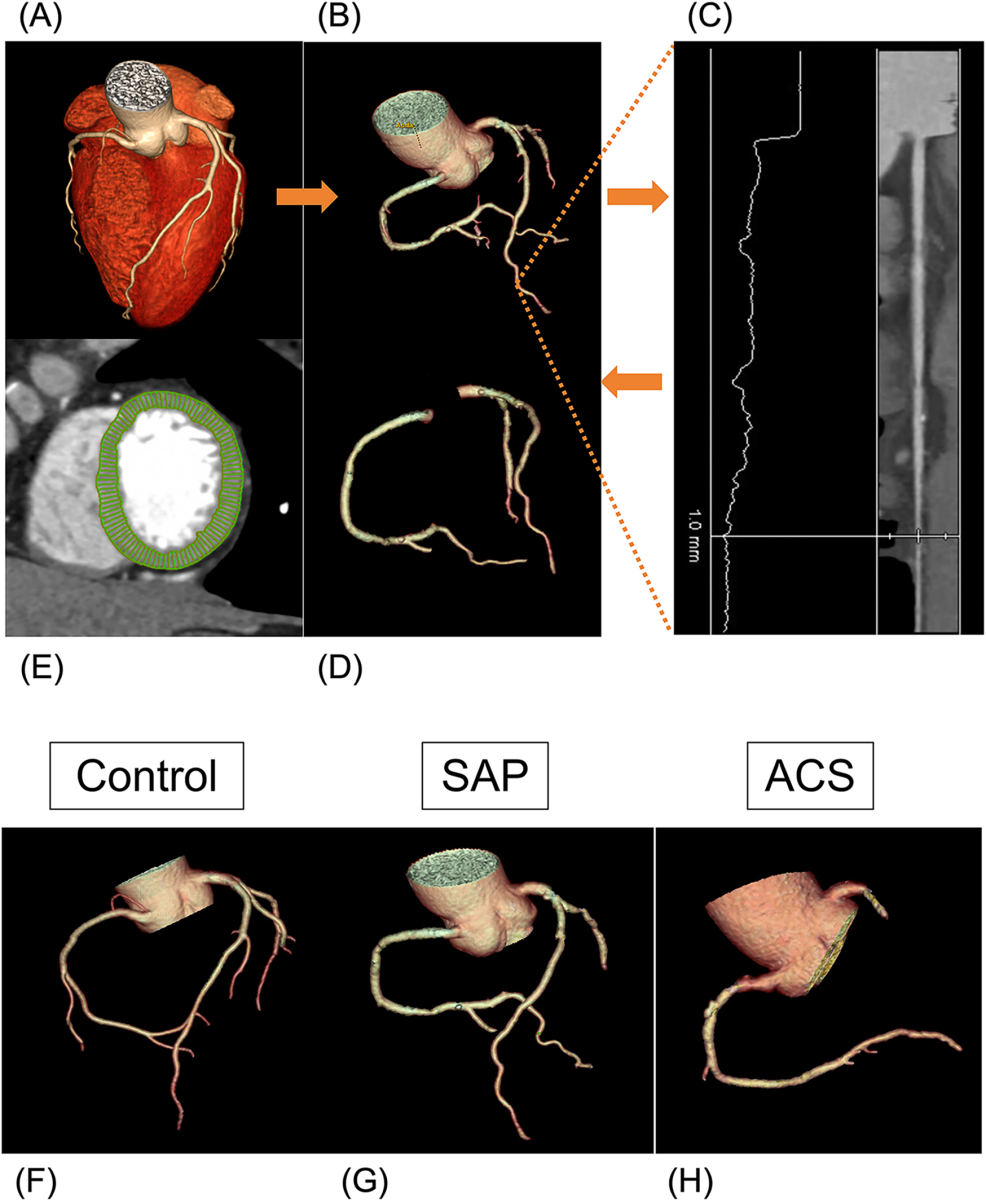
Volume to mass analysis. **(A–D)** Flowchart of obtaining coronary lumen volume (V). **(A)** CCTA image reconstruction. **(B)** Segmentation of the whole arterial tree. **(C)** Defining a 1 mm threshold on the distal end of each vessel in the straightened multiplane reconstructions manually. **(D)** Extraction of the total coronary lumen volume. **(E)** Segmentation of the left ventricle myocardial. **(F–H)** Examples of three arterial tree images acquired with the CCTA reconstruction. **(F)** A 53-year-old woman (V 5,360 mm^3^, V/M 57.7 mm^3^/g) diagnosed with non-CAD. **(G)** A 66-year-old man (V 3,702 mm^3^, V/M 34.6 mm^3^/g) diagnosed with SAP. **(H)** A 63-year-old man (V 3,265 mm^3^, V/M 19.8 mm^3^/g) diagnosed with ACS.

The sequential steps required for V/M analysis have been previously described by Taylor et al. (3). First, the scanned images were transmitted to a dedicated software (AW Version 4.7, GE Healthcare, USA). The whole arterial tree was then segmented and extracted from the imaging data of the x-ray window at 75% of the RR cycle. If necessary, adjustments to the vessel centerline and boundaries were made manually. The reader manually defined a 1 mm threshold on the distal end of each vessel in the straightened multiplane reconstructions (as shown in Figure 1). Consequently, branches of the main epicardial coronary arteries identified in the coronary CTA data > 1 mm in diameter were included, and the total coronary lumen volume was obtained by summating all the segmented coronary arteries (6). For left ventricle myocardial mass, the volume was segmented from CCTA images and multiplied by the myocardial tissue density (1.05 g/ml) to obtain the LV mass, M. Next, the V/M ratio was calculated by dividing the coronary artery volume by the LV myocardial mass. An experienced radiologist (S.D., with 30 years of experience), blinded to clinical parameters, conducted this analysis. To ensure consistency, two radiologists (Y.B. and X.F., with 30 and 20 years of experience, respectively) did a second measurement for 20% of patients one month later, with good intra- and inter-observer reproducibility (ICC = 0.78 and 0.76, respectively).

### 2.4 Semiautomated plaque quantification

**Figure 2:**
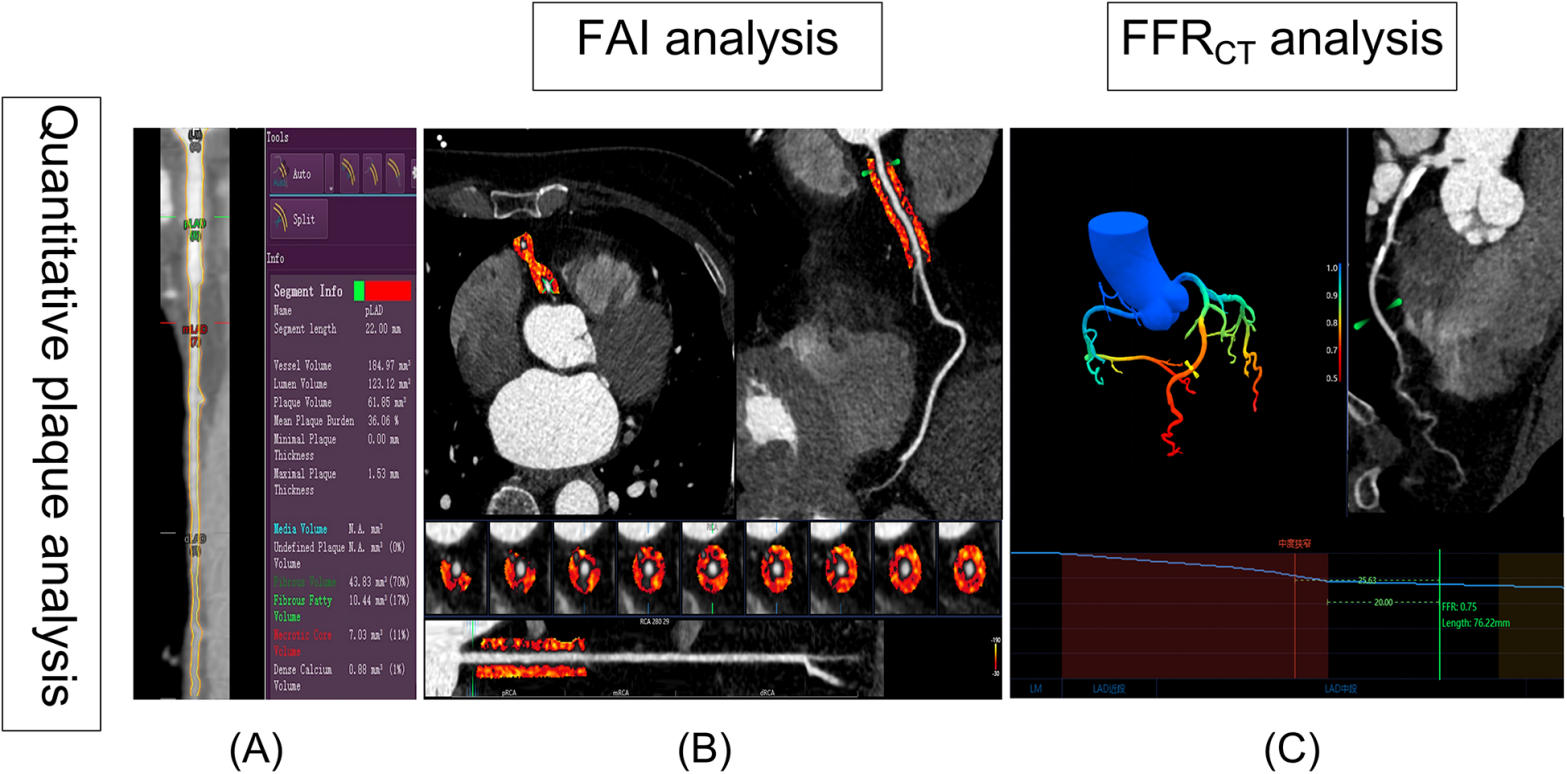
Semiautomated plaque quantification, FAI and FFR_CT_ analysis. **(A)** The red lines represent the automatic lumen segmentation results, while the yellow lines indicate the lumen segmentation results after manual correction. On the right, the results of plaque automatic analysis are visible. **(B)** The FAI was automatically extracted by Skviewer software from the proximal 10–50 mm segment of the right coronary artery (the orange region in B). **(C)** Diagram of FFR_CT_ analysis: The color gradient in the legend represents the FFR_CT_ values ranging from 0.5 to 1.0. The blue line chart depicts the FFR_CT_ values along the LAD. Based on the definition of FFR_CT_, the location of the most significant stenosis is identified (depicted as moderate stenosis in the image), and the FFR_CT_ value is measured at a point 20 mm distal to the stenosis, with FFR_CT_ = 0.75. FAI, fat attenuation index; FFR_CT_, coronary CT angiography-derived fractional flow reserve.

The Digital Imaging and Communications in Medicine files submitted by the site were analyzed for coronary CTA, with clinical results and case status masked. Independent and experienced readers used standardized measurements and semiautomated plaque analysis software (QAngio CT Research Edition version 3.2, Medis Medical Imaging Systems, Leiden, the Netherlands) with appropriate manual correction (8). The semiautomated software reported the following cross-sectional plaque characteristics per plaque analyzed: maximal plaque volume (PV), % Diameter stenosis (% DS), max plaque length (PL), maximal plaque burden (plaque volume divided by vessel volume × 100%, PB), and the proportion of each component of the plaque by composition (calcified, fibrous, fibrofatty, and necrotic core) (Figure 2A).

### 2.5 FFR_CT_ and FAI analysis

In recent years, the peri-coronary fat attenuation index (FAI), which is measured using CCTA images, can be used to reflect peri-coronary inflammation and could improve the prediction of a heart disease risk. The Skviewer software intelligent analysis system (Skviewer; Shukun Technology, Beijing, China) was used to extract FAI automatically. FAI were measured using the method described by Oikonomou et al. (9). The FAI measurement involved analyzing the proximal 10–50 mm of the right coronary artery (RCA) (Figure 2B). Fractional flow reserve obtained from CT-derived fractional flow reserve (FFR_CT_) is an innovative technique that utilizes sophisticated hydrodynamic analytical methods applied to data from CCTA. All FFR_CT_ values were recorded distal to the highest grade/greatest percentage stenosis only in the left anterior descending (LAD) branch for each patient using Skviewer software intelligent analysis system. This value was referred to as the FFR_CT_ value 2 cm distal to the site of the stenosis (Figure 2C) (10). The above measurement results were completed independently by a radiologist (S.D., with 30 years of experience) who were blinded to the clinical details.

### 2.6 Clinical data collection

Clinical data were obtained upon hospital admission. Demographic data, body mass index (BMI), heart rate (HR), systolic blood pressure (SBP), diastolic blood pressure (DBP), laboratory data, and medical history were recorded.

### 2.7 GRACE risk score calculation

The GRACE risk score assessed several predictive factors, including age, HR, SBP, serum creatinine (Cr) level, Killip class, cardiac arrest upon hospital admission, elevated cardiac biomarkers, and ST-segment deviation. ST-segment deviation refers to ST-segment elevations or depressions of at least 1 mm in the anterior, inferior, or lateral leads on electrocardiograms. Killip class was determined by the presence of rales and S3 sounds in the lungs and was classified as I (absent), II (<50% lung fields), III (>50% lung fields), or IV (shock). Elevated cardiac biomarkers were identified by the presence of high-sensitivity troponin I or other cardiac enzymes exceeding the upper reference limit. These factors were evaluated upon admission (11). An experienced cardiologist, Dr. C.X., who has 20 years of experience, recorded the patient's clinical characteristics and calculated the GRACE score.

### 2.8 Statistical analysis

Statistical analyses were conducted in R (version 4.2.3) and SPSS (version 26.0). Continuous variables were presented as mean ± standard deviation (SD) for normal distributions or median with [interquartile range (IQR)] for non-normal distributions. Categorical variables were expressed as frequencies and percentages. The Mann–Whitney U test was used for binary group comparisons, while one-way ANOVA or Kruskal–Wallis test with Bonferroni *post-hoc* analysis was employed for three-group comparisons. Categorical variables were analyzed using the Chi-squared test. Furthermore, the Mantel test, performed using the “ggcor” package in R, evaluated the impact of CCTA features on CAD risk stratification. Pearson analysis was used to examine correlations between V/M and other CCTA features. To ascertain the predictive efficacy of V/M for ACS, parameters such as specificity, sensitivity, and the area under the Receiver Operating Characteristic (ROC) curve were meticulously calculated, and the “pROC” package in R was used to draw the ROC curve. The predictive capability of the models was evaluated by calculating the area under the receiver operating characteristic curve (AUC). The discrepancies in AUC values between various models were evaluated using the De-Long test. The calibration curve was used to assess the predictive performance of various models. Furthermore, the net reclassification index (NRI) was calculated to assess the incremental predictive value of V/M. In the ACS sub-groups analysis, Uni- and multivariate logistic regression analyses were used to identify independent predictors of high-risk GRACE risk score; the correlation between the V/M ratio and GRACE risk score was scrutinized through Pearson analysis. A *p*-value < 0.05 represented statistical significance.

## 3 Results

### 3.1 Clinical data

The final cohort consisted of 450 participants (63 ± 10 years, 65% male), 168 patients diagnosed with ACS, 160 with SAP, and 122 healthy controls. The baseline characteristics of all patients revealed no notable variations in age, BMI, HR, SBP, DBP, total cholesterol (TC), low-density lipoprotein cholesterol (LDL-C), or cardiovascular risk factors (including hypertension, diabetes mellitus, hyperlipidemia, and CAD family history) among the three groups (Table 1). However, it is worth noting that male patients and smokers were more common in the ACS and SAP groups compared to the control group. Additionally, cardiac troponin I (cTnI), brain natriuretic peptide (BNP), fasting glucose (Glu), hemoglobin A1c (HbA1c), Cr, total triglycerides (TG), and lipoprotein (a) levels were notably higher in the ACS and SAP groups than in the control group, while high-density lipoprotein cholesterol (HDL-C) levels were lower in the former groups than in the latter.

**Table 1:** Baseline characteristics for the patient populations.

Variables	ACS (*n* = 168)	SAP (*n* = 160)	Control (*n* = 122)	*P* value
Age (years)	62.83 (±11.59)	63.43 (±9.92)	62.61 (±7.88)	0.776
Sex, male, *n* (%)	129 (77. 8)^a^	102 (63.8)^b^	62 (50.8)^c^	<0.001*
Smoking,n (%)	54 (32.1)^a^	49 (30.6)	15 (12.3)^c^	<0.001*
Hypertension,n (%)	105 (62.5)	96 (60.0)	63 (51.6)	0.164
Diabetes, *n* (%)	58 (34.5)	61 (38.1)	33 (27.0)	0.145
Hyperlipidemias, *n* (%)	39 (23.2)	52 (32.5)	28 (30.0)	0.096
CAD family history, *n* (%)	5 (2.9)	4 (2.3)	4 (2.3)	0.925
Body mass index (kg/m^2^)	25.01 (±3.63)	24.64 (±2.80)	24.36 (±2.26)	0.198
Heat rate (bpm)	78.94 (±13.18)	75.98 (±25.66)	74.54 (±11.26)	0.107
Systolic blood pressure (mmHg)	138.68 (±20.29)	136.14 (±12.41)	132.70 (±32.40)	0.079
Diastolic blood pressure (mmHg)	82.10 (±12.89)	79.84 (±10.07)	79.84 (±9.11)	0.108
cardiac troponin I (*μ*g/L)	2.54 (±7.85)^a^	0.03 (±0.07)^b^	0.01 (±0.01)	<0.001*
Brain natriuretic peptide (ng/L)	800.34 (±3,103.29)^a^	111.75 (±183.80)^b^	20.50 (±26.10)	<0.001*
Fasting glucose (mmol/L)	6.34 (±2.03)^a^	6.23 (±2.35)	5.38 (±0.96)^c^	<0.001*
Hemoglobin A1c (%)	6.80 (±4.51)^a^	6.70 (±1.44)	5.90 (±0.68)	0.020*
Creatinine (μmol/L)	74.44 (±19.86)^a^	72.60 (±19.81)	64.08 (±14.90)^c^	<0.001*
Total cholesterol (mmol/L)	4.48 (±1.26)	4.33 (±1.27)	4.37 (±0.88)	0.496
Total triglycerides (mmol/L)	2.02 (±1.28)^a^	1.94 (±1.78)	1.47 (±0.74)^c^	0.002*
HDL-C (mmol/L)	1.00 (±0.25)^a^	1.10 (±0.37)	1.29 (±1.19)	0.002*
LDL-C (mmol/L)	2.57 (±0.93)	2.47 (±0.99)	2.52 (±0.68)	0.592
Lipoprotein(a) (mmol/L)	0.27 (±0.24)^a^	0.29 (±0.25)	0.18 (±0.21)^c^	0.001*

Values are mean (±SD) or *n* (%).

ACS, acute coronary syndrome; SAP, stable angina pectoris; HDL-C, high-density lipoprotein cholesterol; LDL-C, low-density lipoprotein cholesterol.

^a^ indicates a comparison between the ACS group and the Control group at *P* < 0.05.

^b^ Indicates a comparison between the SAP group and the ACS group at *P* < 0.05.

^c^ Indicates a comparison between the SAP group and the Control group at *P* < 0.05.

* Indicates statistical significance (*P* < 0.05). One-way ANOVA test followed by Bonferroni *post-hoc* test was used for comparisons involving three groups.

### 3.2 Baseline of CCTA features

Supplementary Table S1 displays the CCTA features of the ACS, SAP, and control groups. For most lesions in the left anterior descending artery, the V/M ratio in ACS and SAP was significantly lower than in controls (21.70 ± 6.96, 31.0 ± 9.90 vs. 43.3 ± 11.5 mm^3^/g; *p* < 0.001). And Supplementary Table S2 showed the baseline patient and CCTA findings between UAP and AMI.

### 3.3 Assessment of ACS and SAP

**Figure 3:**
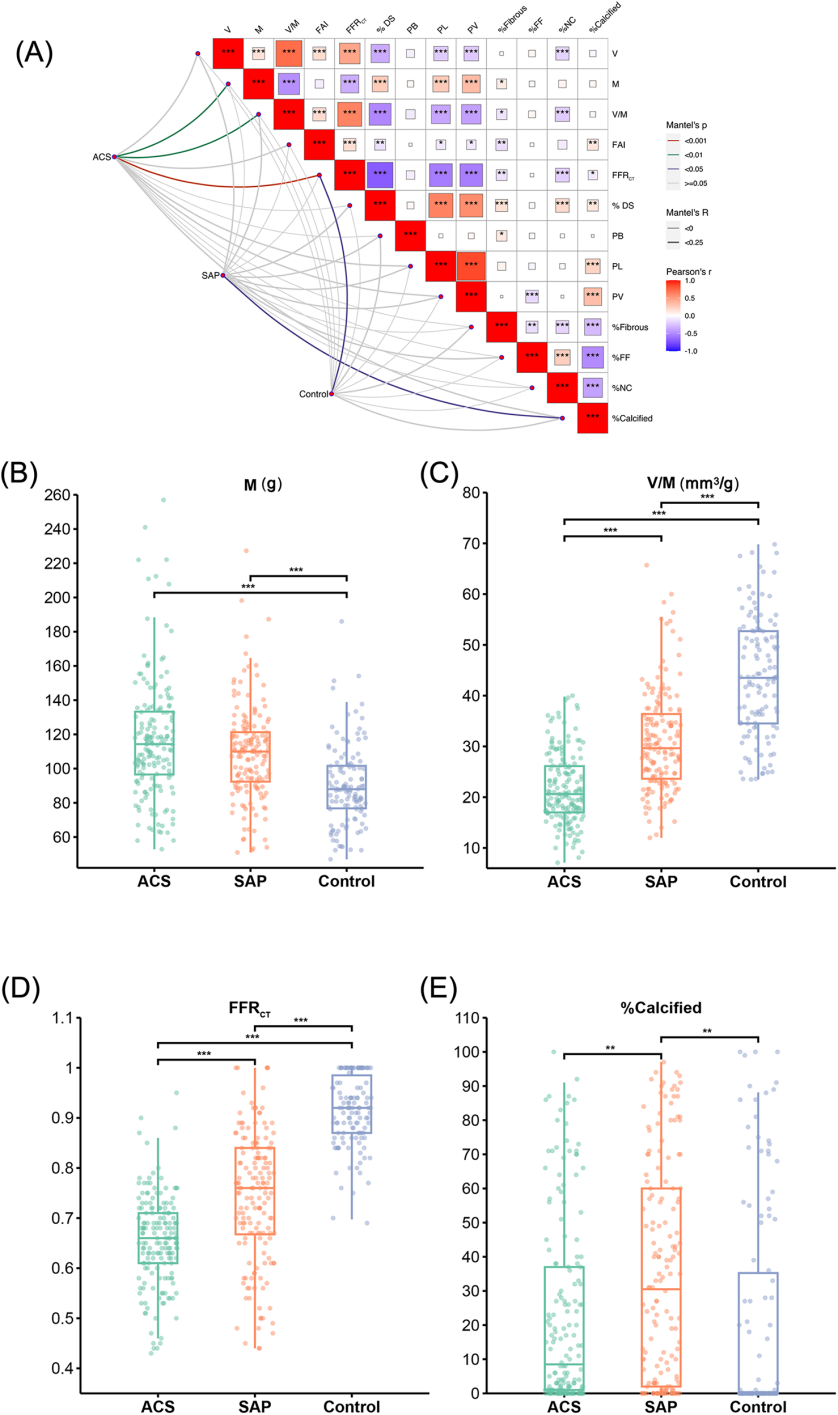
Results of risk stratification influencing factors on patients with suspected CAD. **(A)** Mantel test results of the impact of CCTA image features on the risk stratification among patients with suspected coronary artery disease. The color gradient denotes the direction of the correlation, and the block size indicates the correlation size, ***p < 0.001, **p < 0.01, *p < 0.05. **(B–E)** M, V/M, FFR_CT_ and % calcified in ACS, SAP and the Control group, respectively. V: epicardial coronary artery lumen volume; M: left ventricle myocardial mass; V/M: ratio of coronary arterial volume to left ventricle myocardial mass; FAI, fat attenuation index; FFR_CT_, coronary CT angiography-derived fractional flow reserve; % DS, % Diameter stenosis; PB, plaque burden; PL, plaque length; PV, plaque volume; %Fibrous, proportion of fibrous components in plaque; %FF, proportion of fibrofatty components in plaque; %NC, proportion of necrotic core components in plaque; %Calcified, proportion of calcification components in plaque; ACS, acute coronary syndrome; SAP, stable angina pectoris.

**Figure 4:**
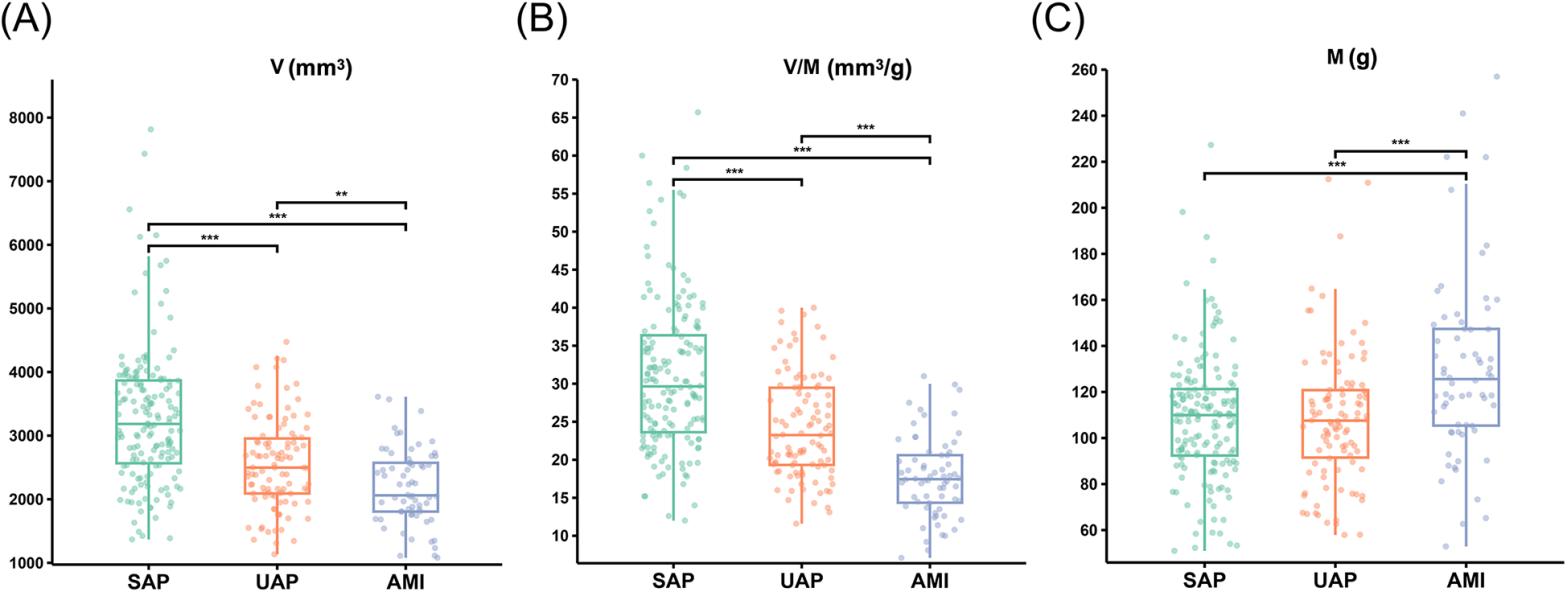
The V, M and V/M in patients with SAP, UAP, and AMI. **(A)** The V in patients with SAP, UAP, and AMI. **(B)** The V/M in patients with SAP, UAP, and AMI. **(C)** The M in patients with SAP, UAP, and AMI. ***p < 0.001 ; **p < 0.01. V: epicardial coronary artery lumen volume; M: left ventricle myocardial mass; V/M: ratio of coronary arterial volume to left ventricle myocardial mass; SAP, stable angina pectoris; UAP, unstable angina pectoris; AMI, acute myocardial infarction.

The Mantel test results shown in Figure 3 revealed that among the cohort with suspected CAD, M, V/M, and FFR_CT_ emerged as pivotal determinants for ACS, with FFR_CT_ having a significant impact (*p* < 0.001). Our findings suggested that FFR_CT_ was the most crucial factor affecting ACS. Moreover, FFR_CT_ in the ACS group was significantly reduced compared to the control and SAP groups. The V/M ratio in ACS was significantly lower than in SAP and controls (21.7 ± 6.96, 31.0 ± 9.90, vs. 43.3 ± 11.50 mm^3^/g; *p* < 0.001); as delineated in Figure 4, a descending trend in V/M ratios was noted, corresponding to the progression of CAD from SAP to UAP to AMI (17.8 ± 5.30, 24.3 ± 6.70, vs. 31.0 ± 9.90 mm^3^/g; *p* < 0.001). Notably, M was slightly higher in coronary heart disease patients (SAP and ACS) than in the control group (117.04 ± 35.20, 109.12 ± 27.56, vs. 90.44 ± 23.36 g; *p* < 0.01). Additionally, a higher proportion of plaque calcification components (%calcified) was predominantly responsible for SAP (*p* < 0.05), and there was no significant difference in the %calcified of plaque components between ACS and the control group. Finally, higher FFR_CT_ was the most significant feature for the control group (*p* < 0.05). A positive correlation was observed between V and M based on Pearson correlation analysis of all enrolled patients. In addition, V/M exhibited a significant positive correlation with FFR_CT_ (*p* < 0.001) and a weakly significant correlation with FAI (*p* < 0.001). However, V/M was negatively correlated with % DS, PL, PV, and %NC (*p* < 0.001). Notably, %NC showed a negative correlation with %Calcified, as illustrated in Figure 3.

### 3.4 Accuracy of V/M to predict acute coronary syndrome

**Figure 5:**
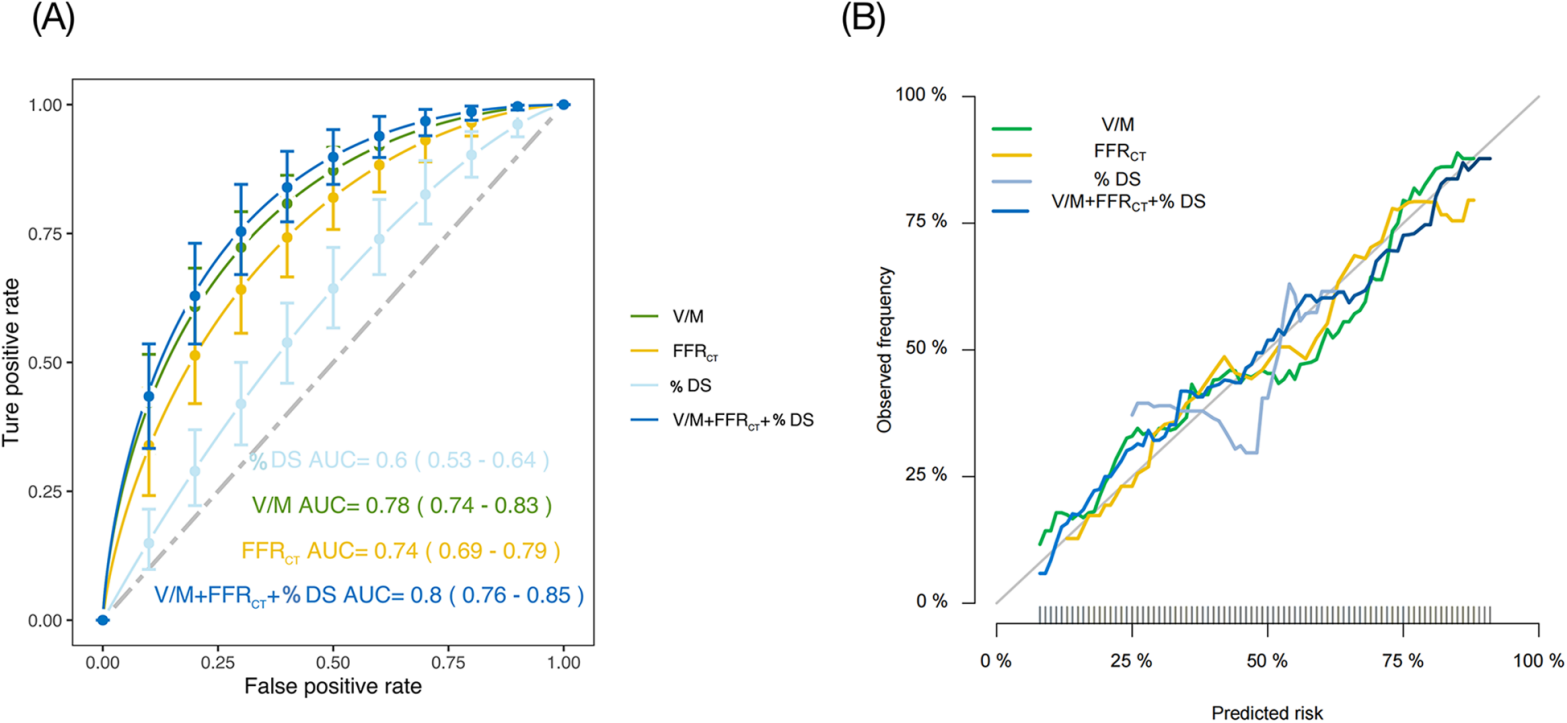
**(A)** ROC of four different models to predict ACS risk status. **(B)** Calibration curves of the four models. % DS, % Diameter stenosis; V/M, ratio of coronary arterial volume to left ventricle myocardial mass; FFR_CT_, coronary CT angiography-derived fractional flow reserve; ACS, acute coronary syndrome; ROC, receiver operating characteristic; AUC, area under curve.

ROC analysis was utilized to examine all continuous CT parameters in the ACS and SAP groups. Following multivariate logistic regression analysis, we created and compared four models to determine ACS risk status: Model 1: % DS, Model 2: V/M, Model 3: FFR_CT_, and Model 4: % DS + V/M + FFR_CT_. Figure 5A illustrated that Model 1 showcased an AUC of 0.60 (95% CI: 0.53 to 0.64), Model 2 showcased an AUC of 0.78 (95% CI: 0.74 to 0.83), while Model 3 displayed an AUC of 0.74 (95% CI: 0.69 to 0.79), and when the three models were combined into a single model, the AUC drastically increased to 0.80 (95% CI: 0.76 to 0.85, *p* < 0.01). The De-Long test revealed that the difference in AUC between Model 4 and Model 2 was not statistically significant (Z = 1.342, *P* = 0.180). However, the AUC of Model 4 was significantly higher than that of Model 1 (Z = 5.292, *P* < 0.001) and Model 3 (Z = 3.252, *P* = 0.001). Additionally, Model 2 demonstrated a higher AUC than both Model 3 (Z = 1.732, *P* = 0.045) and Model 1 (Z = 4.174, *P* < 0.001). The calibration curve indicated that the combined model exhibited a high level of calibration (Figure 5B). Furthermore, when assessed using the net reclassification index (NRI), the combined model improved the predictive ability of V/M by 5.45% (NRI = 0.0545; *P* = 0.166). The predictive ability of FFR_CT_ was enhanced by 13.18% (NRI = 0.1318; *P* = 0.019), and the predictive ability of % DS was improved by 21.49% (NRI = 0.2149; *P* = 0.001).

### 3.5 Baseline of clinical features in the sub-groups of ACS patients

As listed in Supplementary Table S3, notable variations were found in age, diabetes, DBP, cTnI, BNP, Glu, HbA1c, HDL-C, and Killip class on admission in the low–intermediate-risk group and high-risk group.

### 3.6 V/M as a predictor of high GRACE risk score

**Figure 6:**
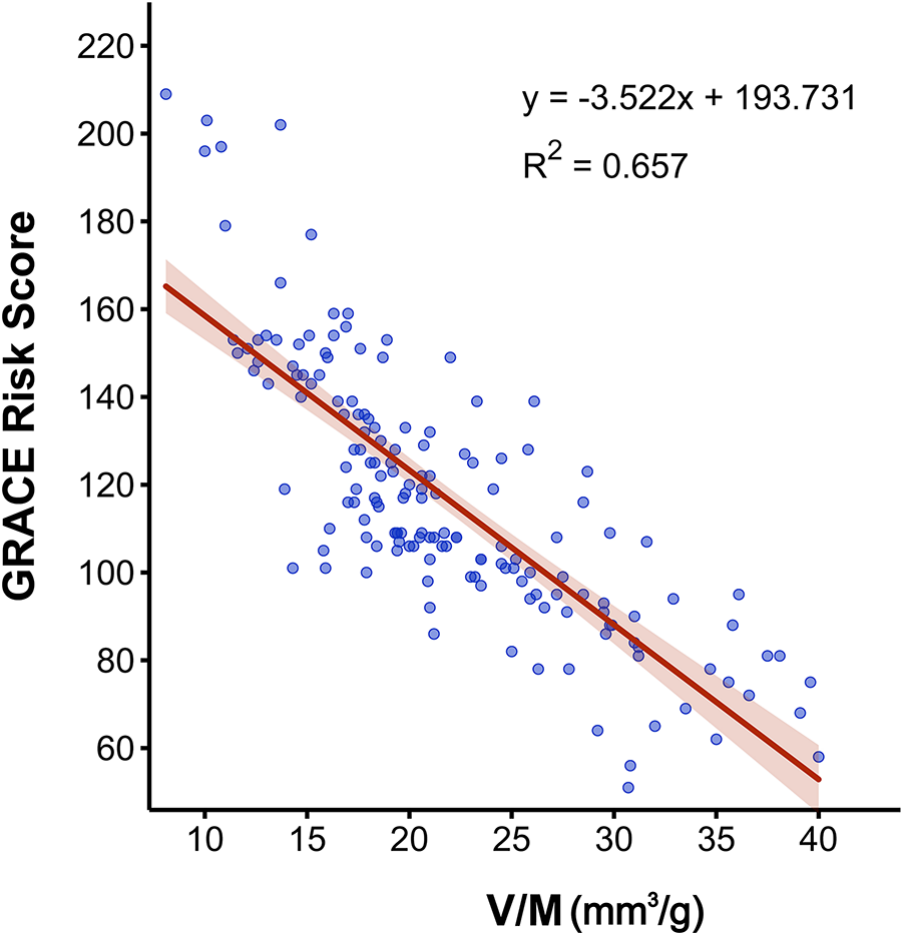
Pearson correlation analysis between V/M and the GRACE risk score in ACS patients. V/M: ratio of coronary arterial volume to left ventricle myocardial mass; GRACE risk score, Global Registry of Acute Coronary Events risk score; ACS, acute coronary syndrome.

As presented in Table 2, univariate analysis showed that several factors, including age, diabetes, DBP, cTnI, BNP, Glu, Cr, LDL-C, V, M, V/M, and proportion of fibrofatty components in plaque (FF%) were all predictors of high GRACE risk score (*p* < 0.05) for both high- and low-intermediate-risk groups. FFR_CT_ did not differ between the high- and low-intermediate-risk groups (*p* = 0.101). A multivariate logistic regression analysis was conducted to ascertain whether V/M was a distinct predictor of high-risk ACS, adjusting for age, V, M, diabetes, DBP, cTnI, BNP, Glu, Cr, LDL-C, and FF%. Results revealed that age and V/M remained independent predictors of high GRACE risk score (*p* = 0.002, 0.024, respectively), with decreased V/M correlating to higher odds of high GRACE risk score. Moreover, the results of Pearson correlation analysis (as shown in Figure 6) showed that the GRACE risk score of ACS patients was significantly negatively correlated with the V/M ratio. The higher the GRACE risk score, the lower the V/M ratio.

**Table 2:** Uni-Multivariate logistic regression analysis for high GRACE risk score in the low–intermediate and high-risk groups.

Variables	Univariate analysis		Multivariate analysis	
	OR (95% CI)	*P* value	OR (95% CI)	*P* value
Clinical features				
Age (years)	1.244 (1.152–1.344)	<0.001*	1.228 (1.081–1.394)	0.002*
Sex, male, *n* (%)	1.244 (0.541–2.858)	0.067	—	—
Smoking, *n* (%)	1.706 (0.743–3.914)	0.208	—	—
Hypertension, *n* (%)	0.832 (0.390–1.775)	0.634	—	—
Diabetes, *n* (%)	2.333 (1.115–4.882)	0.024*	2.179 (0.342–13.875)	0.410
Hyperlipidemias, *n* (%)	0.967 (0.412–2.266)	0.938	—	—
CAD family history	1.078 (0.285–4.082)	0.911	—	—
Body mass index (kg/m^2^)	1.024 (0.927–1.131)	0.645	—	—
Heat rate (bpm)	1.022 (0.995–1.050)	0.112	—	—
Systolic blood pressure (mmHg)	0.986 (0.967–1.004)	0.131	—	—
Diastolic blood pressure (mmHg)	0.958 (0.927–0.989)	0.009*	1.016 (0.964–1.071)	0.553
Cardiac troponin I (μg/L)	1.080 (1.016–1.149)	0.013*	1.115 (0.983–1.265)	0.089
Brain natriuretic peptide (ng/L)	1.001 (1.001–1.002)	<0.001*	1.001 (1.000–1.001)	0.070
Fasting glucose (mmol/L)	1.377 (1.153–1.644)	<0.001*	1.304 (0.809–2.104)	0.276
Hemoglobin A1c (%)	1.019 (1.001–1.037)	0.033*	1.001 (0.963–1.039)	0.974
Creatinine (μmol/L)	0.892 (0.664–1.198)	0.449	—	—
Total cholesterol (mmol/L)	0.853 (0.613–1.185)	0.343	—	—
Total triglycerides (mmol/L)	1.248 (0.289–5.394)	0.767	—	—
HDL-C (mmol/L)	0.542 (0.344–0.854)	0.008*	1.923 (0.744–4.974)	0.177
LDL-C (mmol/L)	2.207 (0.547–8.904)	0.266	—	—
CCTA characteristics				
V (mm^3^)	0.999 (0.998–1.000)	0.002*	1.009 (0.998–1.021)	0.108
M (g)	1.043 (1.026–1.060)	<0.001*	0.893 (0.750–1.064)	0.206
V/M (mm^3^/g)	0.521 (0.411–0.661)	<0.001*	0.153 (0.030–0.785)	0.024*
FAI	0.977 (0.937–1.018)	0.263	—	—
FFR_CT_	0.015 (0.001–0.992)	0.101	—	—
Minimal lumen diameter (%)	1.448 (0.219–9.575)	0.701	—	—
Plaque burden (%)	0.970 (0.801–1.174)	0.752	—	—
Plaque length (mm)	1.000 (0.979–1.022)	0.973	—	—
Plaque volume (mm^3^)	1.002 (1.000–1.005)	0.067	—	—
Percentage of plaque composition				
Fibrous (%)	0.994 (0.979–1.010)	0.488	—	—
Fibrofatty (%)	0.976 (0.954–0.998)	0.036*	1.010 (0.962–1.060)	0.696
Necrotic core (%)	1.015 (0.998–1.033)	0.092	—	—
Calcified (%)	1.006 (0.994–1.019)	0.339	—	—

HDL-C, high-density lipoprotein cholesterol; LDL-C, low-density lipoprotein cholesterol; V, epicardial coronary artery lumen volume; M, left ventricle myocardial mass; V/M, ratio of coronary arterial volume to left ventricle myocardial mass; FFR_CT_, coronary CT angiography-derived fractional flow reserve.

* Indicates statistical significance (*P* < 0.05).

## 4 Discussion

Our main findings indicated that individuals with suspected CAD are at a progressively higher risk of ACS as their V/M ratios decrease. Lower V/M ratios were found with the progression of CAD from SAP to UAP to AMI. CCTA-derived V/M provided incremental predictive value for ACS beyond FFR_CT_ and % DS, thus improving risk stratification. Furthermore, the GRACE risk score of ACS patients was significantly negatively correlated with the V/M ratio and the V/M ratio was a significant predictor of the high GRACE risk score in ACS patients, independently of other factors.

Recent studies have confirmed that cardiac computed tomography-derived coronary artery volume to myocardial mass is feasible for diagnosing coronary artery disease (12–14). The initially proposed ratio of coronary lumen volume to left ventricular mass V/M was in the NeXt sTeps (NXT) trial (3). Talor et al. found that patients with low V/M ratios had more significant diameter stenosis, higher total plaque volume, and lower FFR_CT_ than those with high V/M, which is consistent with our observations. A significant positive correlation was also observed between V/M and FFR_CT_, suggesting that V/M is a quantitative indicator of the imbalance between coronary blood supply and myocardial demand, resulting in a corresponding change in FFR_CT_. Moreover, it should be emphasized that the Mantel test was used to analyze the effect of CCTA image features on the risk stratification of patients with suspected coronary heart disease. Firstly, results showed that for all patients suspected of CAD, M, V/M, and FFR_CT_ were the primary influencing factors for ACS, with FFR_CT_ having a significant impact (*p* < 0.001). The analysis underscored the paramount importance of FFR_CT_ in influencing ACS pathogenesis. Moreover, the V/M ratio in ACS and SAP was significantly lower than in controls (*p* < 0.001), which may be due to an interplay between vascular supply and myocardial hypertrophy, whereby the former lags behind the latter and leads to a decline in V/M. Hypoxia-inducible factor-1- alpha (HIF-1α) is essential for angiogenesis in ischemic tissue (15). It is also a known regulator of endothelial function that could contribute to reduced coronary blood flow reserve, which may, in turn, lead to changes in coronary shear stress and mechanical transduction, thus attenuating arterial generation and driving low V/M (16). In comparison, the left ventricular myocardial mass (M) exhibited a slight increase in coronary heart disease patients (SAP and ACS) than in the controls (*p* < 0.001). The observed elevation in the left ventricular mass among coronary heart disease patients may be attributed to compensatory hypertrophy of the myocardium resulting from prolonged hypoxia. The primary cause of myocardial hypertrophy in patients with CAD is myocardial ischemia. When the coronary blood supply is inadequate, the myocardium receives insufficient oxygen and nutrients, Hypoxia-inducible factor-1α (HIF-1α) has an essential role in ventricular remodeling processes involving myocardial fibrosis and hypertrophy (17), prompting the myocardial cells to undergo physiological hypertrophy as an adaptive response to the hypoxic environment. Prolonged ischemia, however, can induce gradual structural changes within the myocardium, potentially leading to the development of hypertrophic cardiomyopathy. Cardiac hypertrophy is an adaptive response to sustained hemodynamic stress, designed to enhance the heart's contractile force by thickening the cardiomyocytes, thereby maintaining adequate circulation. However, this hypertrophic response is often maladaptive and may be incomplete, potentially progressing to cardiac dysfunction.

In patients with ACS and SAP, it makes sense to focus on the V/M ratio. To the best of our knowledge, only one previous study evaluated V/M in patients with ACS (18). This investigation enrolled patients manifesting ST-segment elevation myocardial infarction (STEMI) post-primary percutaneous coronary intervention, juxtaposing them against a cohort with stable coronary artery disease (CAD) within the NXT trial. Results showed lower median V/M after STEMI (53 vs. 65 mm^3^/g, respectively, *p* = 0.009). This may suggest that the fundamental value of the V/M ratio is reduced, and even after revascularization, the coronary artery blood supply might be inadequate to meet the long-term myocardial demand, thereby elucidating the risk of recurrent AMI post-revascularization. In contrast to the study mentioned above, our research concentrated on the V/M ratio antecedent to the onset of ACS and SAP. We observed a progressive decline in V/M ratios correlating with the advancement of CAD from SAP to UAP to AMI (17.8 ± 5.30, 24.3 ± 6.70, vs. 31.0 ± 9.90 mm^3^/g; *p* < 0.001). This trend instigated a reduction in the epicardial coronary artery volume, potentially attributable to coronary atherosclerosis, plaque progression, and other factors. Consequently, the V/M ratio emerged as a salient tool for risk stratification in ACS, underscoring the need for heightened vigilance towards V/M alterations in patients with presumptive coronary heart disease. Secondly, our study delineated a greater prevalence of %Calcified, which was predominantly responsible for SAP compared to the other groups, whereas no significant disparity was observed between ACS and the control group in this regard. The observation was consistent with prior research by Jinnouchi et al., which investigated the nexus between calcification and plaque stability/instability. The study showed that patients with a history of stable angina were more likely to have fibrous, calcium-enriched plaques, with highly calcified plaques being inherently more stable than their less calcified counterparts (19). The rationale is that acute thrombotic episodes, like plaque rupture and erosion, are the primary catalysts for acute coronary syndromes and occur less frequently in stable, fibrous calcified plaques than in those with lower calcification levels. Our findings also illustrated that ACS patients exhibited a higher proportion of necrotic core components (NC%) than those with SAP, who manifested a higher %Calcified. Hence, a substantial proportion of plaque calcification components may augment plaque stability to a certain degree. In summary, CCTA-derived V/M could distinguish the entire spectrum of CAD patients and potentially curtail unnecessary hospital admissions and invasive angiographic procedures.

CCTA-derived V/M provided incremental predictive value for ACS beyond FFR_CT_ and % DS, improving risk stratification. CCTA has become the most commonly used gatekeeper for patients with coronary heart disease (20). It also has an increasingly important role in patients with ACS. However, its low specificity in identifying functional myocardial ischemia could not manifest functional stenosis in patients with mild lesions and angina pectoris (21). Therefore, integrating anatomical and physiological aspects is crucial for improving the diagnostic capability of CCTA. FFR_CT_ is a newly emerging approach to determine lesions with significant hemodynamic changes based on CCTA images. However, considering that the diagnostic accuracy of the FFR_CT_ value in the grey zone range of 0.63–0.83 is relatively low (22), it is reasonable to find another way to overcome this defect. While FFR_CT_ is the ratio of pressures proximal and distal to the stenosis to assess blood supply, the V/M ratio can quantify the ability of the coronary arteries to supply blood relative to myocardial demand and can, therefore be used as an additional indicator of coronary circulatory function. Our study demonstrated that V/M outperformed FFR_CT_, % DS in predicting ACS [AUC: 0.78 [95% CI: 0.74–0.83] vs. 0.74 [95% CI: 0.69–0.79], 0.60 [95% CI: 0.53–0.64]], and the combined AUC of the three increased significantly, reaching 0.80 [95% (CI): 0.76–0.85]. Given its non-invasive, convenient, and rapid nature, extracting more effective CCTA image features to improve diagnostic performance is an emerging and critical research area.

V/M resulted as an independent predictor of a high GRACE risk score (GRACE risk score >140) in a sub-group analysis of ACS (*p* < 0.001). Patients in the high-risk group had lower V/M than the inter-media risk group. The GRACE risk score was established to predict thrombotic events in patients with ACS. Our analysis indicated a significant moderate inverse relationship between higher GRACE risk scores and lower V/M ratios. These results highlighted the critical role of the V/M ratio as a predictive marker for high-risk ACS cases. Consequently, this suggested its potential utility as a risk stratification tool in managing ACS patients, providing valuable insights for clinical decision-making and treatment strategies.

It is worth highlighting that previous research has found that the volume of the coronary lumen only slightly increased after nitrate-induced coronary vasodilation before CCTA, and there was a significant increase in the coronary artery lumen volume, V, but not in the V/M ratio (23). However, patients in the present study did not need to take nitroglycerin before CCTA, meaning we did not observe the effect on the coronary lumen volume or V/M ratio. Nevertheless, our patients exhibited a higher V/M ratio (30.9 mm^3^/g) than those reported in some mainstream studies, which may be because our study included East Asian individuals, who have been shown to have higher V/M ratios (29.2 mm^3^/g) than other ethnic groups (24). In addition, our healthy control group had a lower degree of coronary atherosclerosis, which could also contribute to the higher V/M results observed in our study cohort.

The present study has several limitations. First, its retrospective design may cause bias in patient selection, though strict criteria were applied to minimize this, and the clinical data of the three groups were well-matched. Second, the quality of the CCTA image acquisition is critical for accurate V/M analysis. Identifying and measuring smaller caliber vessels can be difficult due to spatial resolution limitations, motion artefacts, and inadequate clouding of the coronary arteries. Additionally, the impact of contrast media turbidity on coronary artery volume calculation remains unclear. Second, our study lacked the use of glyceryl trinitrate (GTN) underwent CCTA for the following reasons: radiologists in our country do not have drug prescribing authority for GTN, with guaranteed image quality, GTN was not used prior to the CCTA; and the use of GTN will dilate the coronary arteries, however, we wanted to study the effect of coronary lumen volume, V/M ratio of patients in their normal state. Third, the included subjects who were examined with CCTA had varying time intervals between their examination and the diagnosis of coronary heart disease. While many patients underwent CCTA just days before their hospital diagnosis, some patients may have experienced changes in the progression of atherosclerosis, plaque volume, and other characteristics, potentially introducing bias into the results. Disease progression is a potential factor that increases the likelihood of a positive result. Fourth, the potential impact of medication use on the V/M ratio was not considered. Certain medications, like statins, may affect the progression of coronary atherosclerosis, leading to changes in the size of the coronary lumen. These changes could result in minor variations in the V/M ratio. Finally, this study did not include invasive fractional flow reserve (FFR), so this should be addressed by subsequent studies. Despite these limitations, our conclusion about the V/M ratio being a valuable tool for assessing ACS remains credible, as extensive research supported its use in quantifying the potential physiological imbalance between blood supply and myocardial demand.

## 5 Conclusions

The V/M analysis based on CCTA is feasible, and the V/M ratio is of particular value for stratified risk prediction of ACS and is independently associated with the GRACE risk score. The study indicated a notably lower V/M ratio in cases compared to controls, with a decreasing trend observed from SAP to UAP to AMI. Furthermore, CCTA-derived V/M provides incremental predictive value for ACS beyond FFR_CT_, and their combined use yielded the most robust predictive performance for ACS risk status, improving risk stratification. The V/M ratio may enhance the diagnostic accuracy of CCTA. These insights underscored the potential of V/M in guiding clinical decision-making and optimizing patient care in recent ACS.

## Data Availability

The raw data supporting the conclusions of this article will be made available by the authors, without undue reservation.
