# Supplementary material for: Cardiac computed tomography-derived coronary artery volume to myocardial mass for the prediction of risk stratification for acute coronary syndrome
Source: Front Cardiovasc Med. 2025 Feb 13;12:1449148. doi: 10.3389/fcvm.2025.1449148 (PMC11865212; doi:10.3389/fcvm.2025.1449148)
Supplement: Supplementary file 1 [file Datasheet1.pdf]

Supplementary Material

1.1 Supplementary Tables

Supplementary Table 1. Baseline Lesion Characteristic at CCTA for all the patients

Variables	ACS (n=168)	SAP (n=160)	Control (n=122)	P value
Lesion location				<0.001*
Left anterior descending, n (%)	108 (64.3)	137 (85.6)	52 (56.0)	—
Right coronary artery, n (%)	49 (29.2)	15 (9.4)	29 (31.1)	—
Left circumflex, n (%)	11 (6.5)	8 (5.0)	12 (12.9)	—
Qualitative features				
V (mm^3), ±SD	2410.96 (±689.82)[a]	3284.04 (±1085.58)[b]	3851.90 (±1239.98)[c]	<0.001*
M (g), ±SD	117.04 (±35.20)[a]	109.12 (±27.56)	90.44 (±23.36)[c]	<0.001*
V/M (g/mm^3)	21.7 (±6.96)[a]	31.0 (±9.90)[b]	43.3 (±11.5)[c]	<0.001*
Fat attenuation index	-75 (-68, -82)	-76 (-70, -82)	-78 (-73, -83)[c]	0.026*
FFR_{CT}, ±SD	0.66 (±0.09)[a]	0.76 (±0.10)[b]	0.91 (±0.07)[c]	<0.001*
% Diameter stenosis	82 (74, 90)[a]	74 (55, 85)	15 (6, 25)[c]	<0.001*
Plaque burden (%), ±SD	42.09 (±14.31)[a]	41.23 (±13.27)	18.12 (±16.21)[c]	<0.001*
Plaque length (mm)	21.12 (12.29, 33.07)[a]	23.94 (13.31, 35.63)	3.92 (0.94, 8.67)[c]	<0.001*
Plaque volume (mm^3), ±SD	151.04 (±135.37)[a]	145.58 (±114.89)	14.96 (±19.64)[c]	<0.001*
Percentage of plaque composition				
Fibrous (%)	33.00 (20.00, 54.75)[a]	24.74 (15.00, 44.00)[b]	22.23 (0, 36.83)[c]	0.049*
Fibrofatty (%)	18.00 (8.00, 32)[a]	16.66 (7.16, 33.38)	5.64 (0, 52.44)	0.005*
Necrotic core (%)	11.00 (3.00, 25)[a]	4.80 (1.00, 12.70)	0 (0, 6.22)[c]	<0.001*
Calcified (%)	8.50 (1.00, 37.00)[a]	30.41 (2.08, 60.09)[b]	0 (0, 36.94)[c]	0.024*

If not stated otherwise, values are median (25th; 75th percentile). Asterisk (*) indicates statistical significance (P<0.05). Kruskal-Wallis test followed by Bonferroni post-hoc test was used for comparisons involving three groups.

[a] indicates a comparison between the ACS group and the Control group at P<0.05.

[b] indicates a comparison between the SAP group and the ACS group at P<0.05.

[c] indicates a comparison between the SAP group and the Control group at P<0.05.

ACS: acute coronary syndrome; SAP: stable angina pectoris; V: epicardial coronary artery lumen volume; M: left ventricle myocardial mass; V/M: ratio of coronary arterial volume to left ventricle myocardial mass; FFR_{CT}: coronary CT angiography-derived fractional flow reserve.

Supplementary Table 2. Baseline characteristics of patients with UAP and AMI

Variables	UAP (n=102)	AMI (n=66)	P value
Clinical features			
Age (years)	61.07(±11.76)	65.55(±10.87)	0.014*
Sex, male, n (%)	81 (79.4)	18 (27.3)	0.352
Smoking, n (%)	38 (37.3)	16 (24.2)	0.092
Hypertension, n (%)	63 (61.8)	43 (65.2)	0.871
Diabetes, n (%)	21 (20.6)	17 (25.8)	0.008*
Hyperlipidemias, n (%)	24 (23.5)	15 (22.8)	>0.999
CAD family history, n (%)	8 (7.8)	6 (9.1)	0.782
Body mass index (kg/m^2)	25.10 (±3.79)	24.84 (±3.39)	0.651
Heat rate (bpm)	78.55 (±12.41)	79.55 (±14.37)	0.634
Systolic blood pressure (mmHg)	139.31 (±21.01)	137.70 (±19.26)	0.616
Diastolic blood pressure (mmHg)	83.50 (±12.89)	79.94 (±12.58)	0.080
cardiac troponin I (µg/L)	0.88 (±2.67)	5.10 (±11.67)	0.001

Brain natriuretic peptide (ng/L)	632.86 (±3103.29)	1059.20 (±3055.32)	0.386
Fasting glucose (mmol/L)	6.19 (±2.10)	6.56 (±1.91)	0.243
Hemoglobin A1c (%)	6.35 (±1.12)	7.51 (±7.03)	0.105
Creatinine (μmol/L)	75.13 (±19.86)	73.37 (±19.81)	0.576
Total cholesterol (mmol/L)	4.55 (±1.14)	4.38 (±1.43)	0.376
Total triglycerides (mmol/L)	2.17 (±1.43)	1.78 (±0.97)	0.052
HDL-C (mmol/L)	0.99 (±0.23)	1.02 (±0.27)	0.509
LDL-C (mmol/L)	2.67 (±0.90)	2.40 (±0.96)	0.067
Lipoprotein(a) (mmol/L)	0.26 (±0.24)	0.28 (±0.25)	0.630
CCTA characteristics			
V (mm^3)	2490.71 (±742.14)	2287.70 (±584.27)	0.050
M (g)	113.32 (±31.19)	122.78 (±40.21)	0.089
V/M (mm^3/g)	22.91 (±7.16)	19.89 (±6.27)	0.006*
FAI	-75 (-68, -80)	-73 (-67, -84)	0.531
FFR_{CT}	0.66 (±0.09)	0.67 (±0.10)	0.500
% Diameter stenosis	85 (75, 90)	81 (64, 90)	0.273
Plaque burden (%)	40.62 (±14.26)	44.36 (±14.20)	0.099
Plaque length (mm)	21.52 (13.00, 34.51)	19.70 (11.42, 32.59)	0.398
Plaque volume (mm^3)	148.17 (±138.90)	155.48 (±130.65)	0.734
Percentage of plaque composition			
Fibrous (%)	37.20 (20.00, 54.75)	39.03 (15.00, 44.00)	0.625
Fibrofatty (%)	24.85 (8.00, 32)	16.66 (7.16, 33.38)	0.109

Necrotic core (%)	11.00 (3.00, 25)	4.80 (1.00, 12.70)	0.754
Calcified (%)	8.50 (1.00, 37.00)	30.41 (2.08, 60.09)	0.621

Asterisk (*) indicates statistical significance (P<0.05). HDL-C: high-density lipoprotein cholesterol; LDL-C: low-density lipoprotein cholesterol; V: epicardial coronary artery lumen volume; M: left ventricle myocardial mass; V/M: ratio of coronary arterial volume to left ventricle myocardial mass; FFR_{CT}: coronary CT angiography-derived fractional flow reserve.

Supplementary Table 3. Baseline characteristics of patients with ACS

Variables	low–intermediate risk group (n=130)	high risk group (n=38)	P value
Age (years),	59 (52.0, 67.3)	75 (71.75, 78.25)	<0.001*
Sex, male, n (%)	101 (77.7)	28 (73.7)	0.607
Smoking, n (%)	45 (34.6)	9 (23.7)	0.204
Hypertension, n (%)	80 (61.5)	25 (65.8)	0.634
Diabetes, n (%)	39 (30)	19 (50)	0.023*
Hyperlipidemias, n (%)	30 (23.1)	9 (23.7)	0.938
CAD family history, n (%)	3 (2.2)	2 (5.3)	0.346
Body mass index (kg/m^2), ±SD	24.93 (±3.65)	25.24 (±3.60)	0.570
Heat rate (bpm)	76.50 (69.75, 87.25)	77.50 (77.15, 91.00)	0.293
Systolic blood pressure (mmHg)	138.00 (126, 149)	133.50 (115.00, 148.25)	0.177
Diastolic blood pressure (mmHg)	81.00 (76.00, 90.25)	79.00 (70.00, 87.25)	0.022*
cardiac troponin I (μg/L)	0.034 (0.012, 0.508)	1.372 (0.108, 6.930)	<0.001*
Brain natriuretic peptide (ng/L)	78.45 (37.00, 78.45)	360.35 (202.50, 1865.00)	<0.001*

Fasting glucose (mmol/L)	5.37 (4.89, 6.59)	7.12 (5.59, 8.84)	<0.001*
Hemoglobin A1c (%)	72.15 (62.15, 78.63)	78.15 (53.25, 91.50)	0.049*
Creatinine (μmol/L)	4.40 (3.67, 5.45)	4.01 (3.25, 5.21)	0.229
Total cholesterol (mmol/L)	1.61 (1.22, 2.59)	1.40 (1.17, 2.37)	0.360
Total triglycerides (mmol/L)	0.95 (0.82, 1.14)	0.99 (0.81, 0.96)	0.688
HDL-C (mmol/L)	2.55 (2.02, 3.22)	2.06 (1.56, 2.57)	0.003*
LDL-C (mmol/L)	0.19 (0.10, 0.35)	0.21 (0.13, 0.54)	0.221
Killip class on admission			0.035*
I	35 (92.1)	18 (64.3)	
II	2 (5.3)	4 (14.3)	
III	—	2 (7.1)	
IV	1 (2.6)	4 (14.3)	
Clinical presentation			<0.001*
Unstable angina pectoris, n	102	—	
NSTEMI, n	23	20	
STEMI, n	5	18	
GRACE risk score, ±SD	105.44 (±20.16)	158.84 (±20.92)	<0.001*

If not stated otherwise, data are median (25th; 75th percentile); Asterisk (*) indicates statistical significance (P<0.05). HDL-C: high-density lipoprotein cholesterol; LDL-C: low-density lipoprotein cholesterol; NSTEMI: Non-st segment elevation myocardial infarction; STEMI: St segment elevation myocardial infarction; GRACE risk score: Global Registry of Acute Coronary Events risk score.
